# *Clostridium difficile* Infection in Outpatients, Maryland and Connecticut, USA, 2002–2007

**DOI:** 10.3201/eid1710.110069

**Published:** 2011-10

**Authors:** Jon Mark Hirshon, Angela D. Thompson, Brandi Limbago, L. Clifford McDonald, Michelle Bonkosky, Robert Heimer, James Meek, Volker Mai, Christopher Braden

**Affiliations:** Author affiliations: University of Maryland School of Medicine, Baltimore, Maryland, USA (J.M. Hirshon);; Centers for Disease Control and Prevention, Atlanta, Georgia, USA (A.D. Thompson, B. Limbago, L.C. McDonald, M. Bonkosky, C. Braden);; Yale School of Medicine, New Haven, Connecticut, USA (R. Heimer, J. Meek);; University of Florida, Gainesville, Florida, USA (V. Mai)

**Keywords:** *Clostridium difficile*, diarrhea, outpatient, enteric, pathogen, bacteria, Maryland, Connecticut, United States, dispatch

## Abstract

*Clostridium difficile*, the most commonly recognized diarrheagenic pathogen among hospitalized persons, can cause outpatient diarrhea. Of 1,091 outpatients with diarrhea, we found 43 (3.9%) who were positive for *C. difficile* toxin. Only 7 had no recognized risk factors, and 3 had neither risk factors nor co-infection with another enteric pathogen.

In the United States, ≈375 million episodes of acute diarrhea occur annually (*1*). Among hospitalized persons, toxin-producing *Clostridium difficile* is a primary diarrheagenic pathogen, usually as a consequence of normal bowel flora distortion caused by antimicrobial drug therapy (*2*,*3*). *C. difficile* infection (CDI) complicates and prolongs hospital stays, leading to increases in health care costs, illness, and death. Recent reports suggest increases in community-onset CDI among persons without recent antimicrobial drug treatment or hospitalization. We describe a prospective evaluation of CDI in persons with diarrhea who visited emergency departments (EDs) and ambulatory primary care clinics in Baltimore, Maryland, and New Haven, Connecticut, and identify microbiologic causes and epidemiologic characteristics of diarrhea. This report highlights cases of outpatient CDI, identifies factors associated with infection, and describes molecular strain characterization.

Patients seeking medical attention for community-onset diarrheal illnesses were enrolled from May 2002 through September 2004 in the EDs and ambulatory clinics at Yale–New Haven Hospital (New Haven, CT, USA) and from May 2002 through July 2007 at EDs and clinics affiliated with the University of Maryland (Baltimore, MD, USA) (*4*).

Informed consent for stool sample collection, initial and follow-up patient interviews, and medical records review was obtained from primarily urban and suburban residents, or parents/guardians for minors, who sought treatment for self-identified primary or secondary diarrhea. This research was approved by the institutional review boards at all participating institutions.

Participants were interviewed at outpatient clinics to assess health status, symptoms, and potential exposures to enteric pathogens, and at follow-up to determine the duration of diarrhea, whether treatment was administered, or whether hospitalization resulted from the initial visit. Stool samples collected during the visit or provided within 48 h and kept cool were homogenized and transferred into multiple vials for storage at –80°C.

An outpatient CDI case was defined as in outpatient with diarrhea whose stool was positive for *C. difficile* toxins by enzyme immunoassay (TOX A/B II ELISA; TechLab, Blacksburg, VA, USA). Presumptive non–health care–associated (NHA) CDI was defined by the absence of an overnight stay at an inpatient healthcare facility over the previous month.

Traditional risk factors for CDI that were investigated included antimicrobial drug use within the past month, age >65 years, serious underlying illness/weakened immune system, history of bowel or ulcer surgery, colon disease, previous CDI, and recent hospitalization. Statistical analysis was done by using SAS version 9.2 (SAS Institute, Inc., Cary, NC, USA). All p values reported are 2-sided, with no correction for multiple comparisons; p<0.05 was considered significant.

*C. difficile* toxin–positive stool specimens, in 1-mL aliquots, were shipped frozen to the Centers for Disease Control and Prevention (Atlanta, GA, USA) anaerobe laboratory for culturing by direct inoculation onto cycloserine cefoxitin fructose agar (CCFA) or ethanol shock, followed by CCFA inoculation. Cultures were incubated for 48–72 h at 35°C under anaerobic conditions and examined for characteristic yellow-green fluorescence under long-wave ultraviolet light and CCFA*p*-cresol odor. *C. difficile* colonies were confirmed with indole (negative) and PRO disk (positive; Remel, Lenexa, KS, USA) tests.

Pulsed-field gel electrophoresis was performed on *C. difficile* genomic DNA digested with *Sma*I, and toxinotyping was performed (*5*). Binary toxin was assayed by PCR for *cdtB* (*6*). Deletions in *tcdC* were detected (*7*).

*C. difficile* toxin tests were performed on 1,091/1,197 stool specimens; 43 (3.9%) of these case-patients met the case definition for outpatient CDI. The mean age of these case-patients was 43.7 years (range 4 months–88 years). Outpatient CDI case-patients were younger at Yale because a significantly greater proportion of toxin-positive children were recruited at Yale (45.5%) than at the University of Maryland (15.6%) (p = 0.04). The 43 outpatient CDI case-patients included 5 infants <1 year of age, 5 children 1–18 years of age, 23 adults 19–64 years of age, and 10 adults >65 years of age; 21 were Caucasian, 18 were African American, and 4 were of other or unknown race/ethnicity (22 male and 21 female case-patients).

Most case-patients (36/43, 83.7%) had a recognized underlying risk factor. Twenty-seven (62.8%) had received systemic antimicrobial drugs, including ciprofloxacin, gaitifloxacin, amoxicillin, ampicillin/sulbactam, piperacillin/tazobactam, cefpodoxime, vancomycin, clindamycin, metronidazole, erythromycin, or trimethoprim/sulfamethoxazole within the preceding month; 14 (32.6%) had been hospitalized; and 15 (34.9%) had chronic illnesses or had undergone bowel surgery that potentially affect immune status or gastrointestinal function (Table 1). Two persons, 1 with AIDS and 1 who underwent a previous bowel resection for diverticulitis, had been treated in the past month for CDI. Only 7 (16.3%) patients had NHA-CDI infections without identified risk factors; 3 were infants (<1 year), 1 was a child (1–18 years), 3 were adults (19–64 years), and none were elderly (>65 years) (Table 2).

**Table 1:** Patient risk factors for CDI compared with those of other patients with diarrhea without CDI, Maryland and Connecticut, USA, 2002−2007*

Risk factor	No. (%) patients with CDI, n = 43	No. (%) patients with diarrhea but not CDI, n = 1,048	p value†
Illnesses potentially affecting immune status			
Lupus	1 (2.3)	4 (0.4)	0.06
Cancer under active treatment	3 (7.0)	40 (3.8)	0.30
HIV/AIDS	2 (4.7)	43 (4.1)	0.86
History of organ transplant	2 (4.7)	22 (2.1)	0.26
Chronic obstructive pulmonary disease (on prednisone)	1 (2.3)	NA	NA
Illnesses potentially affecting gastrointestinal function			
Crohn disease	0 (0)	14 (1.3)	0.45
Ulcerative colitis	1 (2.3)	19 (1.8)	0.81
Prior bowel or ulcer surgery	6 (14.0)	69 (6.6)	0.06
Any medical or surgical condition	15 (34.9)	176 (16.8)	0.002
Hospitalized within prior month	14 (32.6)	92 (8.8)	<0.001
Antimicrobial drug therapy within prior month	27 (62.8)	231 (22.0)	<0.001
No hospitalization or antimicrobial drug therapy within prior month and no predisposing condition	7 (16.3)	698 (66.6)	<0.001

*CDI, *Clostridium difficile* infection; NA, not available. †Uncorrected χ^2^.

**Table 2:** Characteristics of CDI case-patients who had no identified risk factors, Maryland and Connecticut, USA, 2002−2007*

Patient ID	Recruitment site	Age/sex	Race/ethnicity	Other medical conditions	Co-infections
1	Maryland	62 y/M	White	Hypertension, GERD, COPD/asthma, depression/anxiety	
2	Maryland	6 mo/M	White	Reflux	*C. perfringens*, rotavirus
3	Yale	20 mo/M	Hispanic	None	Norovirus
4	Yale	5 mo/M	White	None	
5	Yale	28 y/F	Black	None	Rotavirus
6	Yale	34 y/F	Hispanic	Polycystic ovary disease, diabetes, GERD	
7	Yale	4 mo/M	Hispanic	None	Norovirus

*CDI, *Clostridium difficile* infection; GERD, gastroesophageal reflux disease; COPD, chronic obstructive pulmonary disease.

The 43 outpatient CDI case-patients were compared with the other 1,048 persons in which *C. difficile* toxin had not been detected. Persons with CDI were, on average, significantly older than others with diarrhea, 43.7 years vs. 29.2 years, respectively (p<0.01). Outpatient CDI case-patients were more likely than *C. difficile*–negative patients to have medical or surgical conditions (34.9% vs.16.8%, p<0.001), been recently hospitalized (32.6% vs. 8.8%, p<0.001), or to have used antimicrobial drugs (62.8% vs. 22.0%, p<0.001).

Co-infections with other enteric pathogens were common among CDI case-patients, including *C. perfringens* (3), rotavirus (5), norovirus (3), sapovirus (2), and 1 each with hookworm, *Bacillus cereus*, astrovirus, and adenovirus. The likelihood of co-infection was similar in patients with (12/36 [33.3%]) and without (3/7 [42.9%]) risk factors (p>0.1).

Of 43 *C. difficile* toxin–positive stools initially tested, 39 stool samples were submitted to the Centers for Disease Control and Prevention for anaerobic culture and *C. difficile* was isolated from 31 samples. Binary toxin was identified in 12 (38.7%). Pulsed-field gel electrophoresis identified 15 different types (Figure). No associations were found between risk factors, including age, and strain or toxinotype (data not shown).

**Figure:**
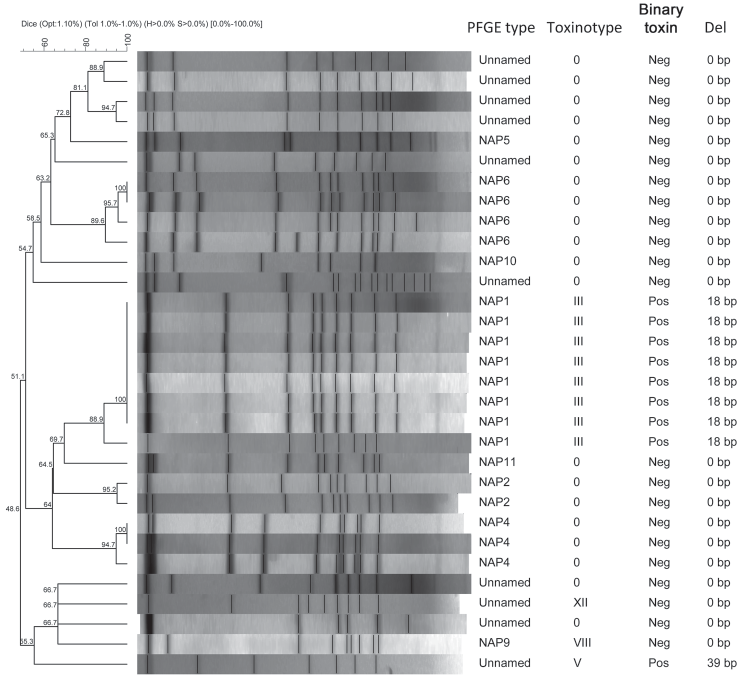
Characteristics of isolates obtained from patients with *Clostridium difficile* infection, Maryland and Connecticut, USA, May 2002–July 2007. PFGE, pulsed-field gel electrophoresis.

NHA-CDI has been recognized for >12 years, and recent reports suggest that disease occurs without patient’s known exposure to antimicrobial drugs or other previously identified risk factors (*2*,*8*–*13*). Although we found a proportion of *C. difficile*–positive diarrheal stools similar to that of 2 other recent prospective studies that used confirmatory culture (i.e., 1.5%–3.9%) for outpatient CDI (*7*,*13*,*14*), we also found a lower proportion of outpatient CDI cases without recognized risk factors of recent hospitalization, chronic medical conditions, recent antimicrobial drug exposure, or co-infection than did those studies.

One limitation of our study was using retrospective self-reporting for assessment of hospitalizations or antimicrobial drug use in the previous month, which potentially can result in recall bias. Also, antimicrobial drug therapy was assessed for only 4 weeks before diarrhea onset; exposure to antimicrobial drugs for a period longer than 1 month before patient seeks treatment may present a risk for CDI. In addition, this study was conducted at 2 urban centers in the eastern United States and may not be generalizable to other locations or clinical settings. Finally, although enzyme immunoassay detection for *C. difficile* was the standard of care at the time of the study, it is now considered too insensitive to be used as a stand-alone diagnostic test (*15*).

In summary, we detected toxigenic *C. difficile* in a similar proportion of patients to those reported in other studies of CDI. However, all but 3 patients had either known risk factors for CDI or other pathogens potentially responsible for their illness; 1 was <1 year of age. *C. difficile* isolates responsible for outpatient CDI are genetically diverse. An evolving picture of widespread, frequent CDI among outpatients without risk factors should be tempered by these findings.
